# A penny for your thoughts: dimensions of self-generated thought content and relationships with individual differences in emotional wellbeing

**DOI:** 10.3389/fpsyg.2013.00900

**Published:** 2013-11-29

**Authors:** Jessica R. Andrews-Hanna, Roselinde H. Kaiser, Amy E. J. Turner, Andrew E. Reineberg, Detre Godinez, Sona Dimidjian, Marie T. Banich

**Affiliations:** ^1^Institute of Cognitive Science, University of Colorado BoulderBoulder, CO, USA; ^2^Department of Psychology and Neuroscience, University of Colorado BoulderBoulder, CO, USA

**Keywords:** mind-wandering, autobiographical, rumination, depression, mindfulness

## Abstract

A core aspect of human cognition involves overcoming the constraints of the present environment by mentally simulating another time, place, or perspective. Although these self-generated processes confer many benefits, they can come at an important cost, and this cost is greater for some individuals than for others. Here we explore the possibility that the costs and benefits of self-generated thought depend, in part, upon its phenomenological *content*. To test these hypotheses, we first developed a novel thought sampling paradigm in which a large sample of young adults recalled several recurring thoughts and rated each thought on multiple content variables (i.e., valence, specificity, self-relevance, etc.). Next, we examined multi-level relationships among these content variables and used a hierarchical clustering approach to partition self-generated thought into distinct dimensions. Finally, we investigated whether these content dimensions predicted individual differences in the costs and benefits of the experience, assessed with questionnaires measuring emotional health and wellbeing. Individuals who characterized their thoughts as more negative and more personally significant scored higher on constructs associated with *Depression *and* Trait Negative Affect*, whereas those who characterized their thoughts as less specific scored higher on constructs linked to *Rumination*. In contrast, individuals who characterized their thoughts as more positive, less personally significant, and more specific scored higher on constructs linked to improved wellbeing (*Mindfulness*). Collectively, these findings suggest that the *content* of people’s inner thoughts can (1) be productively examined, (2) be distilled into several major dimensions, and (3) account for a large portion of variability in their functional outcomes.

## INTRODUCTION

*“You are today where your thoughts have brought you; you will be tomorrow where your thoughts take you.”* – James Allen (1864–1912), author.

A wealth of recent cognitive and neuroscientific research suggests that humans spend a remarkable amount of time engaged in *self-generated thought *(Singer, 1966; Klinger, 1971; Klinger and Cox, 1987; Kane et al., 2007; Killingsworth and Gilbert, 2010; Song and Wang, 2012), an internal mode of cognition characterized by its capacity to overcome the constraints of the external environment (Smallwood, 2013). Supporting its frequent occurrence, self-generated thought is associated with a wide variety of *benefits*, enabling us to confront future challenges, solve problems, and navigate our social world (Smallwood and Schooler, 2006; Baars, 2010; Christoff et al., 2011; McMillan et al., 2013; Mooneyham and Schooler, 2013; Smallwood and Andrews-Hanna, 2013). At the same time, numerous studies suggest the experience can be associated with significant *costs*, including disrupted task performance and poor psychological wellbeing (Smallwood and Schooler, 2006; Moberly and Watkins, 2008; Watkins, 2008; Killingsworth and Gilbert, 2010; McVay and Kane, 2010; Mooneyham and Schooler, 2013; Smallwood and Andrews-Hanna, 2013). In fact, a variety of mental health disorders are defined by impairments arising from self-generated thought (Harvey et al., 2004), and such findings suggest that the adaptive and maladaptive manifestation of the experience varies widely across individuals. Understanding the factors that give rise to this variability could help individuals harness the beneficial aspects of self-generated thought and, in doing so, improve their psychological wellbeing.

Here we tackle this research objective by testing recent hypotheses that the *content* of self-generated thought represents an important factor underlying its costs and benefits (Watkins, 2008; Mar et al., 2012; Smallwood and Andrews-Hanna, 2013). The *content regulation hypothesis* proposes that**self-generated thoughts are particularly beneficial for individuals who are able to regulate the content of the experience to positive or productive topics (Smallwood and Andrews-Hanna, 2013). In line with this hypothesis, we predicted that individuals who primarily engaged in positive or productive thoughts would exhibit improved emotional health and wellbeing, whereas negative or unproductive thought content would be indicative of poor emotional health and wellbeing. Whereas existing studies primarily focus on the frequency of self-generated thought or a single aspect of thought content (i.e. valence) – typically assessed in the context of cued autobiographical memory retrieval paradigms, mood induction paradigms, or ongoing experimental tasks – we examined several aspects of self-generated thoughts that occupy participants’ minds throughout their daily lives. By developing a novel thought sampling paradigm in which participants generate several recurring thoughts and rate each thought on numerous content-specific variables, we were able to (1) quantify normative estimates of thought content in a large group of adults, (2) examine multi-level relationships among content variables, and (3) explore which content variables predicted unique variance in the costs and benefits of the experience.

We focused on individual differences in *depressive symptoms* as an important marker of poor emotional wellbeing because (1) major depressive disorder (MDD) is the leading cause of disability worldwide (World Health Organization, 2008), (2) depressive symptoms predict significant distress and impairment at subclinical levels of severity (Judd et al., 1998), and (3) individuals with a current or past history of depression often exhibit intrusive self-generated thoughts focused on negative self-schema (Beck, 1967; Watkins, 2008). Depressed individuals also “overgeneralize” based on single instances (Beck, 1967) and exhibit overgeneral autobiographical memories that tend to be associated with a broad range of unconstructive consequences (Williams et al., 2007; Watkins, 2008). However, it is neither clear whether overgeneral memory applies to the types of self-generated thoughts that occur in daily life, nor whether these characteristics are driven by the affective symptoms of depression or the repetitive styles of thinking associated with the disorder. To disentangle the effects of negative affect and repetitive thought on the content of self-generated thought, we also examined individual differences in *rumination*, a repetitive style of thinking focused on “one’s symptoms of distress and the circumstances surrounding these symptoms” (Nolen-Hoeksema et al., 1997, 2008; Nolen-Hoeksema, 2000). We hypothesized that individuals with higher levels of depressive symptoms would exhibit thought content characterized as more negative, more self-focused and – to the degree that they *also* ruminate – less specific.

To explore which aspects of thought content predict adaptive functional outcomes, we examined individual differences in *mindfulness* – a purposeful, present-minded, and non-judgmental mode of awareness linked to improved health and emotional wellbeing (Brown and Ryan, 2003; Bishop, 2004). When measured as a trait, mindfulness seems to buffer against symptoms of depression and rumination (Brown and Ryan, 2003), and elements of mindfulness have been incorporated into effective treatment therapies for a variety of mental health disorders, including depression and anxiety (Kabat-Zinn, 1990; Segal et al., 2002; Baer, 2003). Mindful individuals report lower frequencies of negative automatic thoughts and a heightened ability to let go of such thoughts (Frewen et al., 2008; see also Evans and Segerstrom, 2011), and mindfulness-based interventions have been shown to alter the nature of self-generated thoughts in similar ways (Frewen et al., 2008). When applied to chronic depression, these therapies have also shown promise in increasing the specificity and perceived likelihood of achieving individuals’ life goals (Crane et al., 2012). Based on these findings, we predicted that dispositional mindfulness would negatively correlate with depression and rumination, and would be associated with thought content characterized as more positive, less self-focused, and more specific.

## MATERIAL AND METHODS

### PARTICIPANTS

Seventy-six young adults (mean age: 21.6 years; 18–35 years; 38 male) participated in the study for paid compensation. To facilitate interdisciplinary analysis for future studies, participants were recruited from a larger database of individuals from the University of Colorado Boulder and the greater Boulder/Denver community who had previously completed a battery of self-report questionnaires, behavioral tasks, and functional neuroimaging measures in our laboratory. Though participants within this database were previously screened for an absence of a current or previous self-reported psychiatric illness, nearly 30% of participants exhibited scores on the Beck Depression Inventory indicative of mild to moderate depression (see below), consistent with prior findings that depressive symptoms often go undiagnosed and untreated (Eisenberg et al., 2007). All procedures were carried out in accordance with the University of Colorado Boulder’s Institutional Review Board.

### MATERIALS AND PROCEDURE

#### Thought sampling paradigm

In a single session, all participants completed an autobiographical thought sampling paradigm, developed for the present study to quantitatively assess the phenomenological content of a broad range of self-generated thoughts and enable computation of reliable within-subject correlations. Participants recalled 36 different thoughts that had recently been on their mind in daily life, generated a three-word description for each thought, and rated each thought on a variety of different *content variables*, including self-relevance, frequency of occurrence, importance/value, centrality to one’s sense of self-identity, valence, emotional intensity, goal orientation, social orientation, specificity, imagery, perspective taking, duration of the topic or event surrounding the thought, temporal orientation, and certainty that the event for which the thought pertains occurred or will occur (see **Table 1** for precise questions). Both the nature of the instructions and the descriptions provided by participants (i.e., “My College GPA,” “Climb Pike’s Peak,” “Miss My Parents”) suggest that participants primarily generated self-generated thoughts that were independent of external stimuli (i.e., stimulus-independent) and similar in content to that observed during episodes of mind-wandering (Stawarczyk et al., 2011).

**Table 1:** Content variables.

Content rating	Question	Scale
Self-relevance	I consider this thought to be highly self-relevant.	0 to 10, from strongly disagree to strongly agree
Frequency	Lately, it seems that this thought has been on my mind a great deal.	0 to 10, from strongly disagree to strongly agree
Importance/value	The topic of this thought is of great value or importance to me.	0 to 10, from strongly disagree to strongly agree
Centrality	This thought contributes to my sense of self-identity.	0 to 10, from strongly disagree to strongly agree
Valence	My own emotions pertaining to this thought are …	0 to 10, from very negative to very positive
Intensity	The intensity of my emotions pertaining to this thought are …	0 to 10, from not intense at all to very intense
Goal-orientation	This thought involves/involved reaching a particular goal of mine.	0 to 10, from strongly disagree to strongly agree
Social-orientation	I consider this thought to involve/concern other people (i.e., an upcoming date, vacation with friends).	0 to 10, from strongly disagree to strongly agree
Detail/specificity	I would characterize this thought as being tied to a highly detailed and specific event.	0 to 10, from strongly disagree to strongly agree
Imagery	When I experience this thought, my mental imagery is …	1 = Perfectly clear and as vivid as normal vision, 2 = Clear and reasonably vivid, 3 = Moderately clear and vivid, 4 = Vague and dim, 5 = No imagery at all, you only “know” that you were thinking of something
Perspective	When I experience this thought, I develop a mental image in which …	1 = I “see” things from my own perspective (like I would in the real world), 2 = I “see” myself from another perspective, 3 = neither
Duration of topic	This thought concerns an event or a topic that lasted or will likely last …	1 = many years, 2 = many months, 3 = a month, 4 = multiple days, 5 = an entire day, 6 = minutes to hours
Temporal orientation	This event took/will take place…	1 = more than 3 years ago, 2 = within the past 3 years, 3 = within the past 365 days (a year’s time), 4 = within the past 30 days (a month’s time), 5 = within the past 7 days (a week’s time), 6 = yesterday, 7 = earlier today, 8 = later today, 9 = tomorrow, 10 = within the next 7 days (a week’s time), 11 = within the next 30 days (a month’s time), 12 = within the next 365 days (a year’s time), 13 = within the next 3 years, 14 = more than 3 years from now.
Certainty	I am certain that the event has taken or will take place for me	0 to 10, from strongly disagree to strongly agree

*
The question assessing temporal orientation and certainty were only answered for thoughts that pertained to a specific event.
*

#### Questionnaires

To assess individual differences in constructs relevant to mental health, a subset of participants (*N* = 70) completed a variety of self-report questionnaires related to emotional health and wellbeing. Whereas the thought sampling paradigm requires that participants retrieve specific examples of self-generated thoughts and rate those thoughts on a variety of different content variables, the mental health questionnaires assess more general aspects of a participant’s wellbeing including his/her behaviors, feelings, actions, and general styles of thinking. We used confirmatory factor analyses (using maximum likelihood estimation) to isolate three latent constructs defined from a large body of clinical literature:* Depression/Negative Affect*, trait *Rumination*, and trait *Mindfulness *(**Table 2**). The overall fit of these models was estimated using a non-significant χ^2^ and Bentler’s comparative fit index (CFI) greater than 0.95 suggestive of an adequate fit of the data.

**Table 2:** Constructs related to emotional health and wellbeing.

Questionnaire	Subscale	Mean	SD	CFA latent construct	Factor loading
MASQ	Loss of Interest	16.13	4.53	Depression/negative affect	0.34*
MASQ	Low Positive Affect	36.20	8.96	Depression/negative affect	0.37*
BDI	Total Score	8.85	6.08	Depression/negative affect	0.73*
Trait PANAS	Negative	17.92	5.66	Depression/negative affect	0.75*
RRS	Brooding	10.59	3.36	Rumination	0.80*
RRS	Depression-related	24.59	6.46	Rumination	0.97*
RRS	Reflection	10.50	3.68	Rumination	0.57*
RRQ	Rumination	40.69	8.18	Rumination	0.30*
FFMQ	Acting with awareness	24.62	5.51	Mindfulness	0.72*
FFMQ	Describing	26.90	5.99	Mindfulness	0.66*
FFMQ	Non-judging of experience	27.49	5.46	Mindfulness	0.69*
FFMQ	Non-reactivity to inner experience	22.28	3.98	Mindfulness	0.45*
FFMQ	Observing sensations, perceptions, thoughts, feelings	25.73	4.60	Mindfulness	-0.15

CFA, confirmatory factor analysis; MASQ, Mood and Anxiety Symptom Questionnaire; BDI, Beck Depression Inventory, 2^nd^ Edition; PANAS, positive and negative affect schedule; RRS, Ruminative Responses Scale; RRQ, Rumination-Reflection Questionnaire; PSWQ, Penn State Worry Questionnaire; FFMQ, Five Facet Mindfulness Questionnaire; *p < 0.05.

Questionnaires assessing depressive symptoms and trait negative affect included the Beck Depression Inventory, 2nd Edition (BDI-2; Beck et al., 1996), the “Low Positive Affect” and “Loss of Interest” subscales of the Mood and Anxiety Symptom Questionnaire (MASQ; Watson and Clark, 1991; Watson et al., 1995a,b), and the “Negative” subscale of the Trait version of the Positive and Negative Affect Schedule**(PANAS; Watson et al., 1988). All questionnaires loaded significantly (*p* < 0.05) on the *Depression/Negative Affect* latent construct (**Table 2**) and provided a good fit to the data (χ^2^ = 3.45, *df* = 2, CFI = 0.96). Note that there was considerable variability in scores across participants. Using previously recommended cutoff scores for the Beck Depression Inventory (Beck et al., 1996), 71% of participants qualified as minimally depressed (scores 0–11), 22% as mildly depressed (scores 12–18), and 7% as moderately depressed (scores 19–29).

Questionnaires assessing trait rumination included “Brooding,” “Depression-Related,” and “Reflection-Related” subscales of the Ruminative Responses Scale (Roberts et al., 1998; Treynor et al., 2003), and the “Rumination” subscale of the Rumination-Reflection Questionnaire (RRQ; Trapnell and Campbell, 1999). All questionnaires loaded significantly (*p* < 0.05) on the *Rumination* latent construct (**Table 2**) and provided an excellent fit (χ^2^ = 0.63, *df* = 2, CFI = 1).

Questionnaires assessing dispositional mindfulness included all five subscales of the Five Facet Mindfulness Questionnaire (Baer et al., 2006). While the “Acting with Awareness,” “Describing,” “Non-judging of Experience,” and “Non-reactivity to Inner Experience” subscales loaded highly onto the *Mindfulness* construct (*p* < 0.05), the “Observe” subscale loaded weakly onto the construct (*p *= 0.27; **Table 2**), consistent with prior observations (Baer et al., 2006). This model also provided an excellent fit to the data (χ^2^ = 3.49, *df* = 5, CFI = 1).

Participants’ factor scores for the *Depression/Negative Affect*, *Rumination*, and *Mindfulness* latent constructs were designated as the dependent (outcome) variable in subsequent multiple regression analyses to explore relationships with the content of individuals’ typical mind-wandering episodes. To examine the specificity of the relationships between *Depression*, *Rumination*, and thought content, we included questionnaires assessing individual differences in anxious arousal (the “Anxious Arousal” subscale of the MASQ) and anxious apprehension (the Penn State Worry Questionnaire, PSWQ; Meyer et al., 1990). We also assessed state affect immediately prior to the thought sampling paradigm using the “Positive” and “Negative” subscales of the State version of the PANAS questionnaire. Note that while the majority of other questionnaires were acquired in the same session subsequent to the thought sampling paradigm, the MASQ and the PSWQ were acquired as part of the larger study an average of 205 days earlier. As both the MASQ and the PSWQ have high test-retest reliability indicative of stable constructs (Stöber, 1998; Keogh and Reidy, 2000), we decided to include these questionnaires in our analyses.

### STATISTICAL METHOD

#### Part 1: Normative estimates of thought content

The first set of analyses sought to quantify normative estimates of thought content across the full group of 76 participants. To extract mean estimates of each content variable, we first averaged participants’ ratings across the 36 different thoughts for each participant separately. For each of the 12 content variables, we then averaged mean ratings across participants. Participants whose mean rating was more than 2.5 standard deviations above or below the group mean were included in figures for visual purposes only and were not included in calculations of the group mean.

#### Part 2: Identifying major dimensions of thought content

The second set of analyses sought to examine relationships among content variables within participants, and in doing so, identify the major dimensions of thought content. For each participant, we computed the relationship between each pair of content variables across his/her 36 different thoughts (i.e., specificity × self-relevancy, specificity × valence, etc.) using Spearman’s rho. Unlike Pearson’s R, Spearman’s rho does not assume the within-participant data are normally-distributed. Next, we averaged the 12 × 12 correlation matrices across participants to generate a group mean correlation matrix. Correlations were assessed for statistical significance by transforming them to Fisher’s *z* using the formula 0.5 × ln[(1 + *r*)/(1 - *r*)] and conducting one-sample *t*-tests across the group to determine if each correlation was significantly different from zero, using an alpha of *p* < 0.05. Given the large number of statistical tests, we also corrected for multiple comparisons using the Bonferroni method and assessed significance against a corrected alpha of *p* < 0.0008.

To identify major *content dimensions* characterizing participants’ self-generated thoughts, we applied hierarchical clustering analysis on the 12 × 12 group mean correlation matrix by first generating a distance matrix using Euclidean distance. *Content dimension scores* for each participant were extracted by converting his/her mean rating for each content variable to a *z*-score using the group mean, and averaging the standardized content variable ratings pertaining to the same cluster. These scores were then used to explore relationships with constructs relevant to emotional health and wellbeing (see below). Note that questions assessing perspective taking and certainty were excluded from the hierarchical clustering analyses. Perspective taking contained no intrinsic order in its response options, while certainty often yielded bimodal distributions at the within-subject level, a strongly positively skewed distribution at the between-subject level (skewness = -0.97), and was only answered for a subset of questions that pertained to a particular event. These non-normal distributions arose because thoughts pertaining to past events were almost always answered with 100% certainty. All other content variables were normally distributed at the between-subject level, with a skewness between –0.5 and +0.5.

#### Part 3: Relationships with constructs relevant to mental health

Our third objective was to examine relationships between individual differences in thought content and dimensional constructs linked to poor and satisfactory emotional health and wellbeing. In the subset of 70 participants who completed all mental health questionnaires, we ran three multiple linear regression analyses in which the participants’ thought content scores extracted from the hierarchical clustering analysis were entered together as independent predictors to predict participants’ factor scores from each of the *Depression/Negative Affect*, *Rumination*, and *Mindfulness *factors, respectively.

Because rumination and depression are often related, and because both depression and rumination often co-occur with anxiety, we also explored the specificity of each construct’s relationship to self-generated thought by conducting part correlations. This procedure involved (1) removing from the dependent variable (i.e., *Depression/Negative Affect*) variance shared with the questionnaires for which we wished to control (i.e., *Anxious Arousal*, *Anxious Apprehension*, and *Rumination*), and (2) using these standardized residuals as new dependent variables in subsequent multiple regressions with thought content scores as predictor variables.

For each multiple regression, we computed Cook’s Distance, a multivariate measure of the statistical influence of an individual on the regression function (Cook, 1977). Outliers with a Cook’s *D* of > 4/*N* were removed from each analysis (Bollen and Jackman, 1987).

In our final set of analyses, we isolated those aspects of thought content that exhibited significant relationships with emotional health and examined whether these relationships persisted after *additionally* controlling for state affect. Partial correlations were conducted between trait factor scores and each significant content dimension separately, while participants’ “Positive” and “Negative” subscales of the State PANAS questionnaire were entered as covariates of non-interest.

All tests were conducted using two-tailed criteria (*p* < 0.05) and analyses were conducted using a combination of R statistical software (R Development Core Team, 2010), SPSS (v21, IBM), and MPlus (Muthén and Muthén, 1998–2011).

## RESULTS

### PART 1: NORMATIVE ESTIMATES OF THOUGHT CONTENT

**FIGURE 1:**
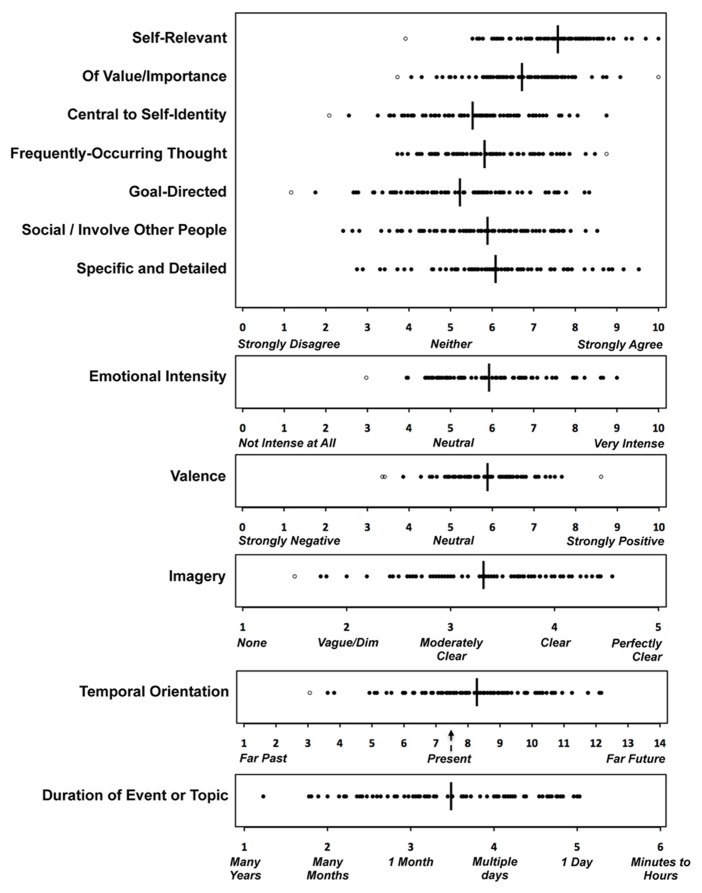
**Phenomenological characteristics of self-generated thought**. Individual data points represent each participant’s mean across 36 self-generated thoughts. Open circles represent outliers > 2.5 SD above or below the group mean (vertical bar) which are not included in calculations. The scale for temporal orientation corresponds to: 1 = more than 3 years ago, 2 = within the past 3 years, 3 = within the past 365 days (a year’s time), 4 = within the past 30 days (a month’s time), 5 = within the past 7 days (a week’s time), 6 = yesterday, 7 = earlier today, 8 = later today, 9 = tomorrow, 10 = within the next 7 days (a week’s time), 11 = within the next 30 days (a month’s time), 12 = within the next 365 days (a year’s time), 13 = within the next 3 years, 14 = more than 3 years from now.

Although the phenomenological characteristics of self-generated thoughts varied widely across participants, several common themes emerged (**Figure 1**). On average, participants characterized their thoughts as highly self-relevant, of moderate importance or value, and moderately central to their sense of self-identity, yet they also indicated their thoughts were moderately social in nature. Participants characterized their thoughts as moderately recurrent, somewhat goal-oriented, of moderate emotional intensity, and positive in valence. Females rated their thoughts as more negative than males; *t*(74) = 2.94, *p* < 0.01. A majority of participants’ thoughts pertained to a particular *event* (mean = 77.4%, SD = 20.0%), although approximately one-quarter did not (mean = 22.6%, SD = 20.0%). Of those thoughts that pertained to a particular event, 59.8% were oriented toward the future (SD = 25.4%) and 40.2% were oriented toward the past (SD = 25.4%). On average, the events for which the thoughts pertained were expected to take place in the *near* future (today or tomorrow).

Participants also characterized their thoughts as exhibiting a moderate degree of specificity and imagery. A first person (“self”) visual perspective was adopted for 64.3% of thoughts (SD = 24.8%), while participants adopted a different perspective for 18.7% of thoughts (SD = 16.0%), and did not adopt a particular perspective for 17.0% of thoughts (SD = 18.8%). On average, participants indicated that their thoughts pertained to topics or events that lasted (or will last) between multiple days and one month in duration.

### PART 2: IDENTIFYING MAJOR DIMENSIONS OF THOUGHT CONTENT

**FIGURE 2:**
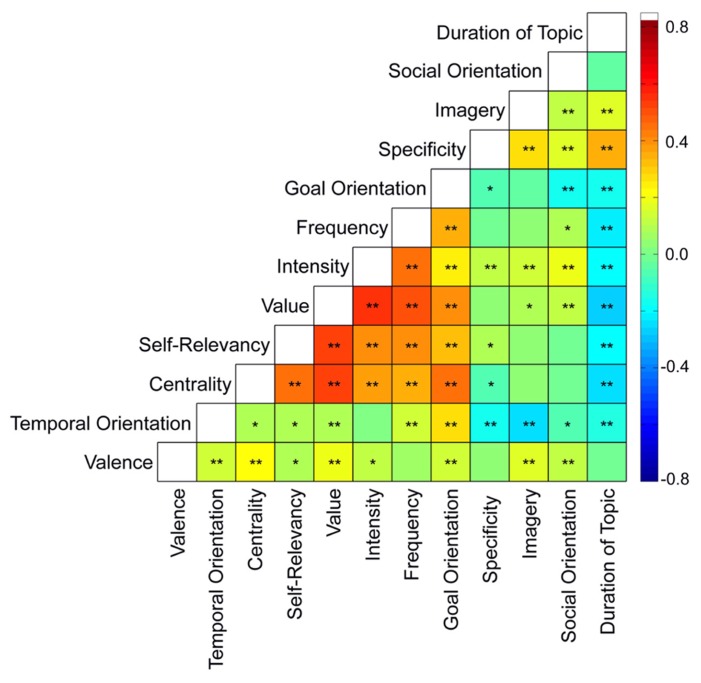
**Relationships between content variables**. Relationships between content variables were averaged across 76 participants. For scales corresponding to each content variable, see **Figure 1** and **Table 1**. Note that higher scores for Duration of Topic correspond to thoughts concerning topics or events lasting *shorter* durations. *p < 0.05, **p < 0.0008 (alpha corrected for multiple comparisons using the Bonferroni method).

**FIGURE 3:**
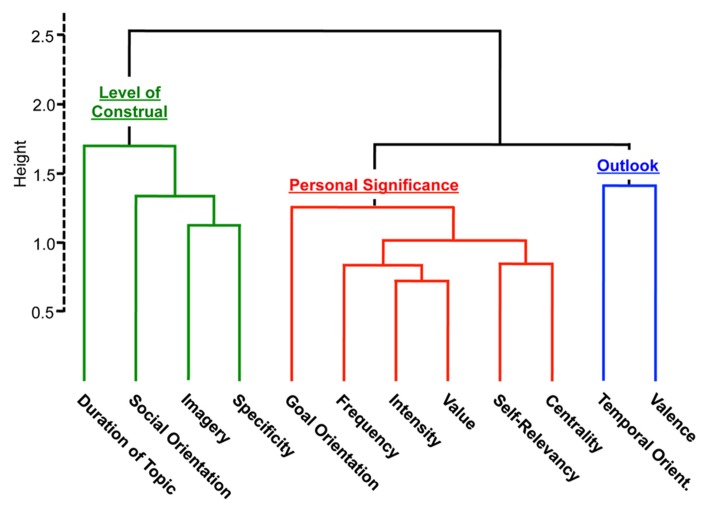
**Hierarchical clustering reveals major dimensions of thought content**. Higher scores for the *Level of Construal* cluster correspond to thoughts that are more temporally and perceptually specific, as well as more socially oriented. Higher scores for the *Personal Significance* cluster correspond to thoughts rated as more personally significant. Higher scores for the *Outlook* cluster correspond to thoughts rated as more positive and future-oriented. On the *y*-axis, height is a measure of similarity or distance between content variables such that two variables forming a single cluster at a small height are more similar than two variables linked at a greater height.

As shown in **Figure 2**, several significant relationships emerged between content variables at the within-subject level, and these relationships are reflected in the pattern of *content dimensions* identified from a hierarchical clustering analysis (**Figure 3**). One dimension, termed *Level of Construal*, refers to the style of abstract versus concrete processing adopted during self-generated thought, and includes questions pertaining to specificity, imagery and duration of the topic or event surrounding the thought. Social orientation also clustered with this dimension. Ratings for questions encompassed in the *Level of Construal* dimension were significantly correlated with one another, with the exception of social orientation (which was only related to specificity and imagery; **Figure 2**). Thoughts rated as more detailed and specific were more likely to pertain to topics characterized by a shorter duration (i.e., a specific episode as opposed to a general topic), to be experienced with more vividness/imagery, and to involve other people.

A second dimension, termed *Personal Significance*, included the following content ratings: self-relevance, centrality, frequency of occurrence, emotional intensity, value/importance, and goal orientation. While all of these questions were significantly positively correlated (all *r’*s > 0.2, *p’*s < 0.001), self-relevance exhibited the strongest relationships with centrality and subjective value (**Figure 2**). Additionally, thoughts that were attributed high value were rated as more central to one’s sense of self-identity, higher in emotional intensity, and more recurrent. Goal-oriented thoughts were also rated as more self-relevant, more central to one’s sense of self-identity, more recurrent, more intense, and more important/valuable.

A third dimension, termed *Outlook*, reflected significant positive relationships between temporal orientation and valence (**Figures 2** and **3**). Future-oriented thoughts were rated as more positive and past-oriented thoughts were rated as more negative.

In addition to relationships between content variables within each content dimension (i.e., self-relevancy, value, etc.), relationships also emerged across variables belonging to different content dimensions (**Figure 2**). More future-oriented thoughts were generally experienced with less specificity, while thoughts that were rated as more personally significant pertained to topics of longer durations, yet were not necessarily less specific or vivid. Furthermore, socially oriented thoughts were experienced with greater emotional intensity, and positive associations were observed between emotional intensity and both imagery and specificity.

### PART 3: RELATIONSHIPS BETWEEN THOUGHT CONTENT AND EMOTIONAL WELLBEING

The combined contribution of the 12 content variables explained a large amount of variance in individual differences in the three psychological constructs of interest. Specifically, thought content explained 45.8% of the variance in *Depression/Negative Affect*, 30.5% of the variance in R*umination*, and 36.3% of the variance in *Mindfulness*. We next repeated the analyses after combining the content variables into the composite dimensions identified in Part 2, but splitting the dimension corresponding to *Outlook* into temporal orientation and valence because of the observed weak correlations between those content ratings and our *a priori *hypotheses linking valence to depression. Thus, four independent variables were entered together into each multiple regression (*Level of Construal*, *Personal Significance*, *Valence, *and *Temporal Orientation*).

**FIGURE 4:**
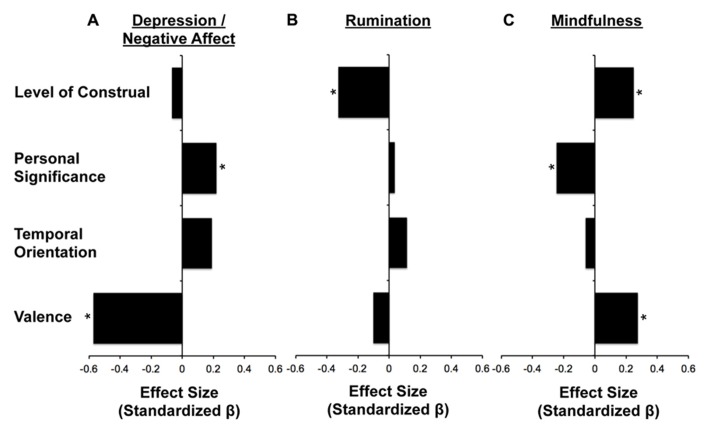
**Independent content predictors of constructs relevant for mental health**. Positive βs are characterized by thoughts that are more concrete, more personally significant, more future oriented and more positive (see scales in **Figure 1**). **(A) ***Personal Significance* and *Valence* contributed a significant amount of variance toward explaining *Depression/Negative Affect*. **(B) ***Level of Construal* was the only dimension that contributed a significant amount of variance toward predicting individual differences in *Rumination*. **(C)** *Level of Construal* and *Personal Significance* contributed a significant amount of variance toward predicting *Mindfulness*, with a considerable additional contribution of *Valence* (β = 0.25, *p* = 0.054). *p ≤ 0.05.

Collectively, these four types of thought content explained 33.0% of the variance in *Depression/Negative Affect*, *R* = 0.58, *F*(4,58) = 7.15, *p* < 0.001, with *Personal Significance* and *Valence* contributing independent variance (*Personal Significance*, β = 0.22, *t*(57) = 1.99, *p* = 0.05; *Valence*, β = -0.57, *t*(57) = -4.65, *p* < 0.001). Individuals who rated their thoughts as more personally significant and more negative reported higher levels of *Depression/Negative Affect* (**Figure 4A**). To examine the specificity of these findings for *Depression/Negative Affect*, we next controlled for statistical effects of anxious arousal, anxious apprehension, and *Rumination* on *Depression/Negative Affect *using a part correlation, and the effects were numerically stronger, *R* = 0.57, *F*(4,56) = 6.20, *p* < 0.001; *Personal Significance*, β = 0.27, *t*(55) = 2.41, *p* < 0.05; *Valence*, β = -0.57, *t*(55) = -4.45, *p* < 0.001.

*Depression Negative Affect *was marginally correlated with *Rumination*, *r*(63) = 0.22, *p* = 0.08, allowing us to examine relationships between self-generated thought content and each mental health construct specifically. Although a smaller percentage of variance in *Rumination *was explained by the four classes of thought content, *R* = 0.38, *F*(4,62) = 2.68, *p* = 0.04, *R*^2^ = 14.7, *Level of Construal* was the only content dimension that contributed significant independent variance to *Rumination*, β = -0.32, *t*(61) = -2.56, *p* < 0.05 (**Figure 4B**). As predicted, individuals who generated more abstract and less specific thoughts were characterized by higher levels of* Rumination*. Importantly, the relationship between *Level of Construal* and *Rumination* became numerically stronger after controlling for comorbid effects of anxiety and *Depression/Negative Affect* on *Rumination*, *R* = 0.41, *F*(4,63) = 3.24, *p* < 0.05, *R*^2^ = 0.17; *Level of Construal:* β = -0.35, *t*(60) = -2.77, *p* < 0.01.

The four types of thought content explained 21.2% of the variance in mindfulness, *F*(4,59) = 3.97, *p* < 0.01, with *Level of Construal, Personal Significance* and *Valence* contributing independent variance (*Level of Construal*, β = 0.25, *t*(58) = 2.00, *p* = 0.05; *Personal Significance*, β = -0.24, *t*(58) = -2.1, *p* < 0.05*; Valence*, β = 0.27, *t*(58) = 2.09, *p* < 0.05). Individuals who rated their thoughts as more concrete and specific, less personally significant, and more positive scored higher on dispositional mindfulness questionnaires (**Figure 4C**). Both *Depression/Negative Affect* and *Rumination* were negatively correlated with *Mindfulness*, *r*(64) = -0.68, *p* < 0.001 and *r*(64) = -0.28, *p* < 0.05.

#### Relationships with state affect

Individual differences in *Depression/Negative Affect* were strongly predictive of state affect as assessed with the “positive” and “negative” subscales of the state PANAS, *R* = 0.59, *F*(2,60) = 15.60, *p* < 0.001. However, state affect was not related to individual differences in *Rumination*, *R* = 0.13, *F*(2,66) = 0.58, *p* = 0.56. State affect was strongly related to *Valence*, *R* = 0.49, *F*(2,69) = 10.73, *p* < 0.001, but not to *Personal Significance*, *R* = 0.12, *F*(2,68) = 0.49, *p* = 0.62. When controlling for state affect, the relationship between *Depression/Negative Affect* and *Valence* remained significant (*r*(62) = -0.51, *p* < 0.001; partial *r*(58) = -0.44, *p* < 0.01).

## DISCUSSION

The current study indicates that self-generated thoughts can be well-characterized along three major content dimensions regarding their level of construal, their personal significance, and the outlook they embody. Moreover, these dimensions explain a notable amount of variance in individual differences in emotional health and wellbeing. As such, our study speaks to a long-standing debate in psychology and neuroscience (e.g., Smallwood and Schooler, 2006; Watkins, 2008; McVay and Kane, 2010) by suggesting that the ability to regulate the *content* of self-generated thought is a key factor underlying its costs and benefits (Smallwood and Andrews-Hanna, 2013). Individuals who characterized their thoughts as more personally significant, more negative, and/or less detailed scored higher on constructs linked to poor psychological wellbeing (*Depression *and *Rumination*), whereas those who characterized their thoughts as less personally significant, more positive, and more specific scored higher on constructs linked to improved wellbeing (*Mindfulness*). Below, we synthesize our findings in the context of prior literature and conclude by discussing future directions for research on self-generated thought and mental health.

### POSITIVE AND PRODUCTIVE MODES OF THOUGHT CONTENT REFLECT THE NORM IN A SAMPLE OF YOUNG ADULTS

Measuring the content of self-generated thought across numerous thought samples, content variables, and participants allowed us to extract at least three dimensions of content: *Level of* *Construal, Personal Significance*, and *Outlook. *On average, participants’ thoughts were moderately perceptually and temporally specific, consistent with prior studies (Klinger and Cox, 1987; Delamillieure et al., 2010; Stawarczyk et al., 2011, 2013). These concrete “low-level” construals have been linked to beneficial outcomes encompassing effective problem solving, successful attainment of future goals, and enhanced self-regulatory behavior (Pham and Taylor, 1999; Watkins and Baracaia, 2002; Watkins and Moulds, 2005; Leary et al., 2006). Interestingly, socially oriented thoughts were also experienced with greater perceptual detail. Although not the main topic of the present manuscript, the finding that participants spend a notable amount of time thinking about other people is in line with prior studies (Mar et al., 2012; see also Ruby et al., under review) and provides strong support for theories highlighting the importance of social information for human cognition (Dunbar, 1998; Baumeister and Masicampo, 2010; Corballis, 2013).

Furthermore, participants’ thoughts were highly personally significant, somewhat goal-oriented, and attributed strong value, resonating with theories that personal concerns form the basis of human thought content (Klinger, 1971, 2009; Fox et al., 2013; McMillan et al., 2013; Stawarczyk et al., 2013).

Consistent with prior studies, participants rated their thoughts as somewhat positive (Killingsworth and Gilbert, 2010; Stawarczyk et al., 2011, 2013; Fox et al., 2013) and future-oriented (Smallwood et al., 2009b; Andrews-Hanna et al., 2010a; Baird et al., 2011; Stawarczyk et al., 2011, 2013; Song and Wang, 2012; Fox et al., 2013; Ruby et al., 2013), supporting a growing body of work regarding the adaptive significance of prospective thoughts (Gilbert and Wilson, 2007; Schacter et al., 2007; Suddendorf and Corballis, 2007). Future-oriented thoughts were perceived as more positive than past-oriented thoughts, supporting prior findings linking retrospective focus to negative mood (Smallwood and O’Connor, 2011).

### THOUGHT CONTENT RELATES TO EMOTIONAL HEALTH AND WELLBEING

Providing strong support for the *content regulation hypothesis* (Smallwood and Andrews-Hanna, 2013), our findings indicate that thought *content* is an important factor underlying the adaptive and maladaptive consequences of self-generated thought. According to *content regulation* *hypothesis*, self-generated thoughts are primarily beneficial when individuals are able to regulate the content of the experience to positive or productive topics (Smallwood and Andrews-Hanna, 2013). Conversely, individuals who lack this capacity may experience maladaptive psychological consequences of self-generated thought.

In line with these predictions, individuals who rated their thoughts as more negative in valence as well as more personally significant scored higher on questionnaires assessing *Depression/Negative Affect*. These findings are consistent with long-held theories that depressed individuals hold negative schemas about themselves, their future, and the environment (Beck, 1967), and that a dispositional style of self-focused thinking exacerbates the duration and symptoms of depression (Smith and Greenberg, 1981; Larsen and Cowan, 1988). Depressed individuals also exhibit attentional and mnemonic biases toward negative and self-referential information, difficulties clearing this information from working memory, and impaired performance particularly when the material is task-irrelevant (Gotlib and Joormann, 2010; Joormann, 2010). Further support for associations between self-focus and negative affect comes from studies linking self-focused attention to state and trait negative affect, particularly when the content of the self-focused thought is negative in nature (Smith and Greenberg, 1981; Mor and Winquist, 2002). Consequently, experimental inductions that amplify external focus and dampen internal focus help depressed individuals adopt a less pessimistic view of their future (Pyszczynski et al., 1987).

Although depressed individuals commonly exhibit overgeneral autobiographical memories (Williams et al., 2007; Watkins, 2008), the present results demonstrate that overgeneral styles of thinking were more strongly related to individual differences in *Rumination* than to *Depression/Negative Affect.* These findings suggest that the repetitive nature of self-generated thought rather than negative affect *per se*, may be most strongly linked to abstract levels of construal. However, since many of the questions contributing to the rumination questionnaires pertain to negative content, it remains yet to be determined whether overgeneral patterns of thought also characterize other types of repetitive thought, independent of affective content. Rumination is strongly related to, yet distinct from, depression, and our observation that overgeneral thinking is particularly related to *Rumination *agrees well with studies reporting elevated overgeneral memory following rumination induction (Watkins and Teasdale, 2001; Sutherland and Bryant, 2007). The relationship between overgeneral memory, rumination, and other types of repetitive thought marks an interesting area for future research.

### MINDFUL ASPECTS OF THOUGHT CONTENT ARE RECIPROCALLY RELATED TO DEPRESSION AND RUMINATION

In contrast to depression and rumination, dispositional mindfulness and mindfulness meditation have been linked to improved health, cognitive ability, and psychological wellbeing (Brown and Ryan, 2003; Sedlmeier et al., 2012). Paralleling these findings, *Mindfulness* exhibited negative relationships with *Depression *and *Rumination*, and was partly explained by an opposing pattern of thought content, being characterized as more positive, less personally significant, and more specific. These results are consistent with prior research demonstrating that mindful individuals report higher levels of positive affect (Brown and Ryan, 2003), positive or less negative thought content (Frewen et al., 2008; Evans and Segerstrom, 2011), and better emotion regulation ability (Farb et al., 2012).

Of relevance, brief mindfulness inductions have been shown to dampen negative affect (Brown and Ryan, 2003; Huffziger et al., 2013), and many of the content variables that predicted increases in dispositional mindfulness in our study are central to mindfulness-based therapies for a variety of mental health disorders. For example, a key element of mindfulness-based cognitive therapy involves teaching people to become aware of the nature of their internal thoughts, view their thoughts from a more distant or “de-centered” perspective, and accept them for what they are rather than the literal truth (Segal et al., 2002; Mennin and Fresco, 2013). When applied to recurrent depression, MBCT reduces relapse rates by nearly 50% (Teasdale et al., 2000; Watkins, 2008) and increases the specificity of individuals’ autobiographical memory (Williams et al., 2000, 2007).

### CONCLUSIONS AND FUTURE DIRECTIONS

In summary, these findings provide strong evidence that the content of people’s inner thoughts in daily life can be productively examined and distilled into several major dimensions. Moreover, individual differences in thought content explain a notable amount of variance in constructs relevant to emotional health and wellbeing. As such, the present manuscript extends previous studies that focus solely on the *frequency *with which self-generated thoughts occur, particularly because increases in frequency have been linked to both adaptive and maladaptive outcomes (i.e., McVay and Kane, 2009; Levinson et al., 2012). In contrast, the *content* characterizing self-generated thought may more precisely constrain the functional consequences of this human experience. Quantifying an individual’s full profile of thought content – perhaps in *combination* with the frequency with which self-generated thoughts occur, and/or the difficulty of the task during which unrelated thoughts occurs (Smallwood and Andrews-Hanna, 2013) – might ultimately help researchers or clinicians distinguish between *different* trait dimensions and/or mental health disorders.

Unfortunately, we were unable to fully address this last question using our sample of participants because none of our participants were diagnosed with a mental health disorder. While we anticipate similar yet stronger findings in individuals with MDD, clinical diagnosis with depression may be associated with patterns of thought content distinct from those highlighted in our non-clinical sample. Although none of our participants reported current or previous diagnosis of depression, they exhibited a broad range of subclinical depressive symptoms, consistent with recent mental health initiatives highlighting the importance of dimensional classification systems (Kupfer and Regier, 2011; Adam, 2013). Furthermore, the finding that specific patterns of thought content predict symptoms of distress even in this non-clinical sample could provide important insight regarding risk factors for the development of more severe symptoms. Biases in thought content may emerge prior to the onset of a major depressive episode, perhaps providing a marker of clinical risk and a target for preventive therapies (Muñoz et al., 2010; Beshai et al., 2011). Future longitudinal research could address these possibilities by assessing changes in thought content over onset and recovery from MDD.

Another open question involves understanding the directional nature of the relationship between thought content and psychological wellbeing. Does dispositional unhappiness exert a causal influence on negative and personally significant thoughts, or does the act of entertaining such thoughts exacerbate poor emotional wellbeing? Might mental health and thought content dynamically influence each other, creating positive feedback loops? While some studies have found initial evidence for causal relationships between the frequency and/or content of self-generated thoughts and wellbeing (Moberly and Watkins, 2008; Smallwood et al., 2009a; Killingsworth and Gilbert, 2010; Smallwood and O’Connor, 2011; Marchetti et al., 2012a; Ruby et al., 2013), a complete understanding of the nature of these relationships will require overcoming several methodological and conceptual challenges (Mason et al., 2013).

Another avenue for future research involves delineating the neural underpinnings of different dimensions of thought content. The brain’s “default network” is a large-scale brain system hypothesized to support spontaneous and goal-directed forms of self-generated thought (Buckner et al., 2008; Andrews-Hanna, 2012). Though our results agree well with findings that depression and rumination are linked to hyperactivity of the default network and increased connectivity with limbic regions (Marchetti et al., 2012b; Whitfield-Gabrieli and Ford, 2012), recent studies suggest that the DMN is organized into multiple subsystems with dissociable functions (Andrews-Hanna et al., 2010b; Andrews-Hanna, 2012). How the content dimensions of self-generated thought map onto these unique DMN components remains an open question.

## Conflict of Interest Statement

The authors declare that the research was conducted in the absence of any commercial or financial relationships that could be construed as a potential conflict of interest.

## References

[B1] Adam D. (2013). On the spectrum. *Nature* 496 416–418 10.1038/496416a 23619674

[B2] Andrews-Hanna J. R. (2012). The brain’s default network and its adaptive role in internal mentation. *Neuroscientist* 18 251–270 10.1177/1073858411403316 21677128 PMC3553600

[B3] Andrews-Hanna J. R. Reidler J. S. Huang C. Buckner R. L. (2010a). Evidence for the default network’s role in spontaneous cognition. *J. Neurophysiol.* 104 322–335 10.1152/jn.00830.2009 20463201 PMC2904225

[B4] Andrews-Hanna J. R. Reidler J. S. Sepulcre J. Poulin R. Buckner R. L. (2010b). Functional-anatomic fractionation of the brain’s default network. *Neuron* 65 550–562 10.1016/j.neuron.2010.02.005 20188659 PMC2848443

[B5] Baars B. J. (2010). Spontaneous repetitive thoughts can be adaptive: postscript on “mind wandering.” *Psychol. Bull.* 136 208–210 10.1037/a0018726 20192560

[B6] Baer R. A. (2003). Mindfulness training as a clinical intervention. *Psychol. Sci.* 10 125–143 10.1093/clipsy.bpg015

[B7] Baer R. A. Smith G. T. Hopkins J. Krietemeyer J. Toney L. (2006). Using self-report assessment methods to explore facets of mindfulness. *Assessment* 13 27–45 10.1177/1073191105283504 16443717

[B8] Baird B. Smallwood J. Schooler J. W. (2011). Back to the future: autobiographical planning and the functionality of mind-wandering. *Conscious. Cogn.* 20 1604–1611 10.1016/j.concog.2011.08.007 21917482

[B9] Baumeister R. F. Masicampo E. J. (2010). Conscious thought is for facilitating social and cultural interactions: how mental simulations serve the animal-culture interface. *Psychol. Rev.* 117 945–971 10.1037/a0019393 20658859

[B10] Beck A. (1967). *Depression: Clinical, Experimental, and Theoretical Aspects*. Philadelphia, Pennsylvania: University of Pennsylvania Press

[B11] Beck A. T. Steer R. A. Ball R. Ranieri W. F. (1996). Comparison of Beck depression inventories-IA and-II in psychiatric outpatients. *J. Pers. Assess.* 67 588–597 10.1207/s15327752jpa6703_13 8991972

[B12] Beshai S. Dobson K. S. Bockting C. L. H. Quigley L. (2011). Relapse and recurrence prevention in depression: current research and future prospects. *Clin. Psychol. Rev.* 31 1349–1360 10.1016/j.cpr.2011.09.003 22020371

[B13] Bishop S. R. (2004). Mindfulness: a proposed operational definition. *Clin. Psychol. Sci. Pract.* 11 230–241 10.1093/clipsy.bph077

[B14] Bollen K. A. Jackman R. W. (1987). “Regression diagnostics: an expository treatment of outliers and influential cases,” in *Modern Methods of Data Analysis,* Vol. 13 eds Fox J. Long J. S. (Newbury Park, CA: Sage) 257–291

[B15] Brown K. W. Ryan R. M. (2003). The benefits of being present: mindfulness and its role in psychological wellbeing. *J. Pers. Soc. Psychol.* 84 822–848 10.1037/0022-3514.84.4.822 12703651

[B16] Buckner R. L. Andrews-Hanna J. R. Schacter D. L. (2008). The brain’s default network: anatomy, function, and relevance to disease. *Ann. N. Y. Acad. Sci.* 1124 1–38 10.1196/annals.1440.011 18400922

[B17] Christoff K. Gordon A. Smith R. (2011). “The role of spontaneous thought in human cognition,” in *Neuroscience of Decision Making* eds Vartanian O. Mandel D. (New York City: Psychology Press) 259–284

[B18] Cook R. D. (1977). Detection of influential observation in linear regression. *Technometrics* 19 15–18 10.2307/1268249

[B19] Corballis M. C. (2013). Wandering tales: evolutionary origins of mental time travel and language. *Front. Psychol.* 4: 1–8 10.3389/fpsyg.2013.00485 23908641 PMC3725404

[B20] Crane C. Winder R. Hargus E. Amarasinghe M. Barnhofer T. (2012). Effects of mindfulness-based cognitive therapy on specificity of life goals. *Cognit. Ther. Res.* 36 182–189 10.1007/s10608-010-9349-4 PMC3348486 22661794

[B21] Delamillieure P. Doucet G. Mazoyer B. Turbelin M.-R. Delcroix N. Mellet E. (2010). The resting state questionnaire: an introspective questionnaire for evaluation of inner experience during the conscious resting state. *Brain Res. Bull.* 81 565–573 10.1016/j.brainresbull.2009.11.014 20003916

[B22] Dunbar R. I. M. (1998). The social brain hypothesis. *Evolut. Anthropol.* 6 178–190 10.1002/(SICI)1520-6505(1998)6:5<178::AID-EVAN5>3.0.CO;2-8

[B23] Eisenberg D. Golberstein E. Gollust S. E. (2007). Help-seeking and access to mental health care in a university student population. *Med. Care* 45 594–601 10.1097/MLR.0b013e31803bb4c1 17571007

[B24] Evans D. R. Segerstrom S. C. (2011). Why do mindful people worry less? *Cogn. Ther. Res.* 35 505–510 10.1007/s10608-010-9340-0

[B25] Farb N. A. Anderson A. K. Segal Z. V. (2012). The mindful brain and emotion regulation in mood disorders. *Can. J. Psychiatry* 57 70–77 22340146 10.1177/070674371205700203 PMC3303604

[B26] Fox K. C. R. Nijeboer S. Solomonova E. Domhoff G. W. Christoff K. (2013). Dreaming as mind wandering: evidence from functional neuroimaging and first-person content reports. *Front. Hum. Neurosci.* 7: 1–18 10.3389/fnhum.2013.00412 23908622 PMC3726865

[B27] Frewen P. A. Evans E. M. Maraj N. Dozois D. J. A. Partridge K. (2008). Letting go: mindfulness and negative automatic thinking. *Cogn. Ther. Res.* 32 758–774 10.1007/s10608-007-9142-1

[B28] Gilbert D. T. Wilson T. D. (2007). Prospection: experiencing the future. *Science* 317 1351–1354 10.1126/science.1144161 17823345

[B29] Gotlib I. H. Joormann J. (2010). Cognition and depression: current status and future directions. *Annu. Rev. Clin. Psychol.* 6 285–312 10.1146/annurev.clinpsy.121208.131305 20192795 PMC2845726

[B30] Harvey A. Watkins E. Mansell W. Shafran R. (2004). *Cognitive Behavioural Processes across Psychological Disorders: A Transdiagnostic Approach to Research and Treatment*. New York City: Oxford University Press

[B31] Huffziger S. Ebner-Priemer U. Eisenbach C. Koudela S. Reinhard I. Zamoscik V. (2013). Induced ruminative and mindful attention in everyday life: an experimental ambulatory assessment study. *J. Behav. Ther. Exp. Psychiatry* 44 322–328 10.1016/j.jbtep.2013.01.007 23466521

[B32] Joormann J. (2010). Cognitive inhibition and emotion regulation in depression. *Curr. Dir. Psychol. Sci.* 19 161–166 10.1177/0963721410370293

[B33] Judd L. L. Akiskal H. S. Maser J. D. Zeller P. J. Endicott J. Coryell W. (1998). Major depressive disorder: a prospective study of residual subthreshold depressive symptoms as predictor of rapid relapse. *J. Affect. Disord.* 50 97–108 10.1016/S0165-0327(98)00138-4 9858069

[B34] Kabat-Zinn J. (1990). *Full-Catastrophe Living: Using the Wisdom of Your Body and Mind to Face Stress, Pain, and Illness*. New York: Bantam Dell

[B35] Kane M. J. Brown L. H. McVay J. C. Silvia P. J. Myin-Germeys I. Kwapil T. R. (2007). For whom the mind wanders, and when: an experience-sampling study of working memory and executive control in daily life. *Psychol. Sci.* 18 614–621 10.1111/j.1467-9280.2007.01948.x 17614870

[B36] Keogh E. Reidy J. (2000). Exploring the factor structure of the Mood and Anxiety Symptom Questionnaire (MASQ). *J. Pers. Assess.* 74 106–125 10.1207/S15327752JPA740108 10779936

[B37] Killingsworth M. A. Gilbert D. T. (2010). A wandering mind is an unhappy mind. *Science* 330 932 10.1126/science.1192439 21071660

[B38] Klinger E. (1971). *Structure and Functions of Fantasy*. New York: John Wiley

[B39] Klinger E. (2009). “Daydreaming and fantasizing: thought flow and motivation,” in *Handbook of Imagination and Mental Stimulation* eds Markman K. D. Klein W. M. P. Suhr J. A. (New York City: Psychology Press, Taylor & Francis Group) 225–239

[B40] Klinger E. Cox W. (1987). Dimensions of thought flow in everyday life. *Imagin. Cogn. Pers.* 7 105–128 10.2190/7K24-G343-MTQW-115V

[B41] Kupfer D. J. Regier D. A. (2011). Neuroscience, clinical evidence, and the future of psychiatric classification in DSM-5. *Am. J. Psychiatry* 168 672–674 10.1176/appi.ajp.2011.11020219 21724672

[B42] Larsen R. J. Cowan G. S. (1988). Internal focus of attention and depression: a study of daily experience. *Motiv. Emot.* 12 237–249 10.1007/BF00993113

[B43] Leary M. R. Adams C. E. Tate E. B. (2006). Hypo-egoic self-regulation: exercising self-control by diminishing the influence of the self. *J. Pers.* 74 1803–1831 10.1111/j.1467-6494.2006.00429.x 17083667

[B44] Levinson D. B. Smallwood J. Davidson R. J. (2012). The persistence of thought: evidence for a role of working memory in the maintenance of task-unrelated thinking. *Psychol. Sci.* 23 375–380 10.1177/0956797611431465 22421205 PMC3328662

[B45] Mar R. A. Mason M. F. Litvack A. (2012). How daydreaming relates to life satisfaction, loneliness, and social support: the importance of gender and daydream content. *Conscious. Cogn.* 21 401–407 10.1016/j.concog.2011.08.001 22033437

[B46] Marchetti I. Koster E. H. W De Raedt R. (2012a). Mindwandering heightens the accessibility of negative relative to positive thought. *Conscious. Cogn.* 21 1517–1525 10.1016/j.concog.2012.05.013 22726693

[B47] Marchetti I. Koster E. H. W. Sonuga-Barke E. J De Raedt R. (2012b). The default mode network and recurrent depression: a neurobiological model of cognitive risk factors. *Neuropsychol. Rev.* 22 229–251 10.1007/s11065-012-9199-9 22569771

[B48] Mason M. F. Brown K. Mar R. A. Smallwood J. (2013). Driver of discontent or escape vehicle: the affective consequences of mindwandering. *Front. Psychol.* 4: 1–12 10.3389/fpsyg.2013.00477 23898317 PMC3722495

[B49] McMillan R. L. Kaufman S. B. Singer J. L. (2013). Ode to positive constructive daydreaming. *Front. Psychol.* 4: 1–9 10.3389/fpsyg.2013.00626 24065936 PMC3779797

[B50] McVay J. C. Kane M. J. (2009). Conducting the train of thought: working memory capacity, goal neglect, and mind wandering in an executive-control task. *J. Exp. Psychol. Learn. Mem. Cogn.* 35 196–204 10.1037/a0014104 19210090 PMC2750806

[B51] McVay J. C. Kane M. J. (2010). Does mind wandering reflect executive function or executive failure? Comment on Smallwood and Schooler (2006) and Watkins (2008). *Psychol. Bull.* 136 188–197; discussion 198–207 10.1037/a0018298 20192557 PMC2850105

[B52] Mennin D. S. Fresco D. (2013). What, me worry and ruminate about DSM-V and RDoC?: the importance of targeting negative self-referential processing. *Clin. Psychol.* 1–22 10.1111/cpsp.12038 PMC5120250 27890972

[B53] Meyer T. J. Miller M. L. Metzger R. L. Borkovec T. D. (1990). Development and validation of the Penn State Worry Questionnaire. *Behav. Res. Ther.* 28 487–495 10.1016/0005-7967(90)90135-6 2076086

[B54] Moberly N. J. Watkins E. R. (2008). Ruminative self-focus and negative affect: an experience sampling study. *J. Abnorm. Psychol.* 117 314–323 10.1037/0021-843X.117.2.314 18489207 PMC2672047

[B55] Mor N. Winquist J. (2002). Self-focused attention and negative affect: a meta-analysis. *Psychol. Bull.* 128 638–662 10.1037/0033-2909.128.4.638 12081086

[B56] Mooneyham B. W. Schooler J. W. (2013). The costs and benefits of mind-wandering: a review. *Can. J. Exp. Psychol.* 67 11–18 10.1037/a0031569 23458547

[B57] Muñoz R. F. Cuijpers P. Smit F. Barrera A. Z. Leykin Y. (2010). Prevention of major depression. *Annu. Rev. Clin. Psychol.* 6 181–212 10.1146/annurev-clinpsy-033109-132040 20192789

[B58] Muthén L. K. Muthén B. O. (1998–2011). *Mplus User’s Guide* 6th Edn Los Angeles, CA: Muthén and Muthén

[B59] Nolen-Hoeksema S. (2000). The role of rumination in depressive disorders and mixed anxiety/depressive symptoms. *J. Abnorm. Psychol.* 109 504–511 10.1037/0021-843X.109.3.504 11016119

[B60] Nolen-Hoeksema S. Mcbride A. Larsen J. (1997). Rumination and psychological distress among bereaved partners. *J. Pers. Soc. Psychol.* 72 855–862 10.1037/0022-3514.72.4.855 9108698

[B61] Nolen-Hoeksema S. Wisco B. E. Lyubomirsky S. (2008). Rethinking rumination. *Perspect. Psychol. Sci.* 3 400–424 10.1111/j.1745-6924.2008.00088.x 26158958

[B62] Pham L. B. Taylor S. E. (1999). From thought to action: effects of process- versus outcome-based mental simulations on performance. *Pers. Soc. Psychol. Bull.* 25 250–260 10.1177/0146167299025002010

[B63] Pyszczynski T. Holt K. Greenberg J. (1987). Depression, self-focused attention, and expectancies for positive and negative future life events for self and others. *J. Pers. Soc. Psychol.* 52 994–1001 10.1037/0022-3514.52.5.994 3585706

[B64] R Development Core Team. (2010). *R: A language and environment for statistical computing. Vienna*. Austria: R Foundation for Statistical Computing Available at: .

[B65] Roberts E. Gilboa E. Gotlib I. H. (1998). Ruminative response style and vulnerability to episodes of dysphoria: gender, neuroticism, and episode duration. *Therapy* 22 401–423

[B66] Ruby F. J. M. Smallwood J. Engen H. Singer T. (2013). How self-generated thought shapes mood – the relation between mind-wandering and mood depends on the socio-temporal content of thoughts. *PLoS ONE* 8:e77554 10.1371/journal.pone.0077554 PMC3806791 24194889

[B67] Schacter D. L. Addis D. R. Buckner R. L. (2007). Remembering the past to imagine the future: the prospective brain. *Nature* 8 657–661 10.1038/nrn2213 17700624

[B68] Sedlmeier P. Eberth J. Schwarz M. Zimmermann D. Haarig F. Jaeger S. (2012). The psychological effects of meditation: a meta-analysis. *Psychol. Bull.* 138 1139–1171 10.1037/a0028168 22582738

[B69] Segal Z. V. Williams J. M. G. Teasdale J. D. (2002). *Mindfulness-Based Cognitive Therapy for Depression: A New Approach to Preventing Relapse*. New York City: Gilford Press

[B70] Singer J. L. (1966). *Daydreaming: An Introduction to the Experimental Study of Inner Experience*. New York: Random House, Inc

[B71] Smallwood J. (2013). Distinguishing how from why the mind wanders: a process-occurence framework for self generated thought. *Psychol. Bull.* 139 519–535 10.1037/a0030010 23607430

[B72] Smallwood J. Andrews-Hanna J. R. (2013). Not all minds that wander are lost: the importance of a balanced perspective on the mind-wandering state. *Front. Psychol.* 4:441 10.3389/fpsyg.2013.00441 PMC3744871 23966961

[B73] Smallwood J. Fitzgerald A. Miles L. K. Phillips L. H. (2009a). Shifting moods, wandering minds: negative moods lead the mind to wander. *Emotion* 9 271–276 10.1037/a0014855 19348539

[B74] Smallwood J. Nind L O’Connor R. C. (2009b). When is your head at? An exploration of the factors associated with the temporal focus of the wandering mind. *Conscious. Cogn.* 18 118–125 10.1016/j.concog.2008.11.004 19121953

[B75] Smallwood J O’Connor R. C. (2011). Imprisoned by the past: unhappy moods lead to a retrospective bias to mind wandering. *Cogn. Emot.* 25 1481–1490 10.1080/02699931.2010.545263 21432633

[B76] Smallwood J. Schooler J. W. (2006). The restless mind. *Psychol. Bull.* 132 946–958 10.1037/0033-2909.132.6.946 17073528

[B77] Smith T. W. Greenberg J. (1981). Depression and self-focused attention. *Motiv. Emot.* 5 323–331 10.1007/BF00992551

[B78] Song X. Wang X. (2012). Mind wandering in Chinese daily lives–an experience sampling study. *PLoS ONE* 7:e44423 10.1371/journal.pone.0044423 PMC3434139 22957071

[B79] Stawarczyk D. Cassol H D’Argembeau A. (2013). Phenomenology of future-oriented mind-wandering episodes. *Front. Psychol.* 4: 1–12 10.3389/fpsyg.2013.00425 23882236 PMC3712143

[B80] Stawarczyk D. Majerus S. Maj M. Van der Linden M D’Argembeau A. (2011). Mind-wandering: phenomenology and function as assessed with a novel experience sampling method. *Acta Psychol.* 136 370–381 10.1016/j.actpsy.2011.01.002 21349473

[B81] Stöber J. (1998). Reliability and validity of two widely-used worry questionnaires: self-report and self-peer convergence. *Pers. Individ. Dif.* 24 887–890 10.1016/S0191-8869(97)00232-8

[B82] Suddendorf T. Corballis M. C. (2007). The evolution of foresight: What is mental time travel, and is it unique to humans? *Behav. Brain Sci.* 30 299–313; discussion 313–351 10.1017/S0140525X07001975 17963565

[B83] Sutherland K. Bryant R. A. (2007). Rumination and overgeneral autobiographical memory. *Behav. Res. Ther.* 45 2407–2416 10.1016/j.brat.2007.03.018 17506978

[B84] Teasdale J. D. Segal Z. V. Williams J. M. G. Ridgeway V. A. Soulsby J. M. Lau M. A. (2000). Prevention of relapse/recurrence in major depression by mindfulness-based cognitive therapy. *J. Consult. Clin. Psychol.* 68 615–623 10.1037/0022-006X.68.4.615 10965637

[B85] Trapnell P. D. Campbell J. D. (1999). Private self-consciousness and the five-factor model of personality: distinguishing rumination from reflection. *J. Pers. Soc. Psychol.* 76 284–304 10.1037/0022-3514.76.2.284 10074710

[B86] Treynor W. Gonzalez R. Nolen-Hoeksema S. (2003). Rumination reconsidered: a psychometric analysis. *Therapy* 27 247–259

[B87] Watkins E. R. (2008). Constructive and unconstructive repetitive thought. *Psychol. Bull.* 134 163–206 10.1037/0033-2909.134.2.163 18298268 PMC2672052

[B88] Watkins E. Baracaia S. (2002). Rumination and social problem solving in depression. *Behav. Res. Ther.* 40 1179–1189 10.1016/S0005-7967(01)00098-5 12375726

[B89] Watkins E. Moulds M. (2005). Distinct modes of ruminative self- focus: impact of abstract versus concrete rumination on problem solving in depression. *Emotion* 5 319–328 10.1037/1528-3542.5.3.319 16187867

[B90] Watkins E. Teasdale J. D. (2001). Rumination and overgeneral memory in depression: effects of self-focus and analytic thinking. *J. Abnorm. Psychol.* 110 353–357 10.1037/0021-843X.110.2.333 11358029

[B91] Watson D. Clark L. A. Tellegen A. (1988). Development and validation of brief measures of positive and negative affect: the PANAS scales. *J. Pers. Soc. Psychol.* 54 1063–1070 10.1037/0022-3514.54.6.1063 3397865

[B92] Watson D. Weber K. Assenheimer J. S. Clark L. A. Strauss M. E. McCormick R. A. (1995a). Testing a tripartite model: I. Evaluating the convergent and discriminant validity of anxiety and depression symptom scales.* J. Abnorm. Psychol.* 104 3–14 10.1037/0021-843X.104.1.3 7897050

[B93] Watson D. Clark L. A. Weber K. Assenheimer J. S. Strauss M. E. McCormick R. A. (1995b). Testing a tripartite model: II. Exploring the symptom structure of anxiety and depression in student, adult, and patient samples. *J. Abnorm. Psychol.* 104 15–25 10.1037/0021-843X.104.1.15 7897037

[B94] Watson D. Clark L. A. (1991). *The Mood and Anxiety Symptom Questionnaire*. Iowa City, IA: University of Iowa

[B95] Whitfield-Gabrieli S. Ford J. M. (2012). Default mode network activity and connectivity in psychopathology. *Annu. Rev. Clin. Psychol.* 8 49–76 10.1146/annurev-clinpsy-032511-143049 22224834

[B96] Williams J. M. G. Barnhofer T. Crane C. Herman D. Raes F. Watkins E. (2007). Autobiographical memory specificity and emotional disorder. *Psychol. Bull.* 133 122–148 10.1037/0033-2909.133.1.122 17201573 PMC2834574

[B97] Williams J. M. G. Teasdale J. D. Segal Z. V. Soulsby J. (2000). Mindfulness-based cognitive therapy reduces overgeneral autobiographical memory in formerly depressed patients. *J. Abnorm. Psychol.* 109 150–155 10.1037/0021-843X.109.1.150 10740947

[B98] World Health Organization. (2008). *The Global Burden of Disease 2004 Update*. Available at:

